# Supplementary material for: HiDeF: identifying persistent structures in multiscale ‘omics data
Source: Genome Biol. 2021 Jan 7;22:21. doi: 10.1186/s13059-020-02228-4 (PMC7789082; doi:10.1186/s13059-020-02228-4)
Supplement: Supplementary file 1 — Additional file 1: Supplementary figures 1-9. [file 13059_2020_2228_MOESM1_ESM.pdf]

Supplementary Figures

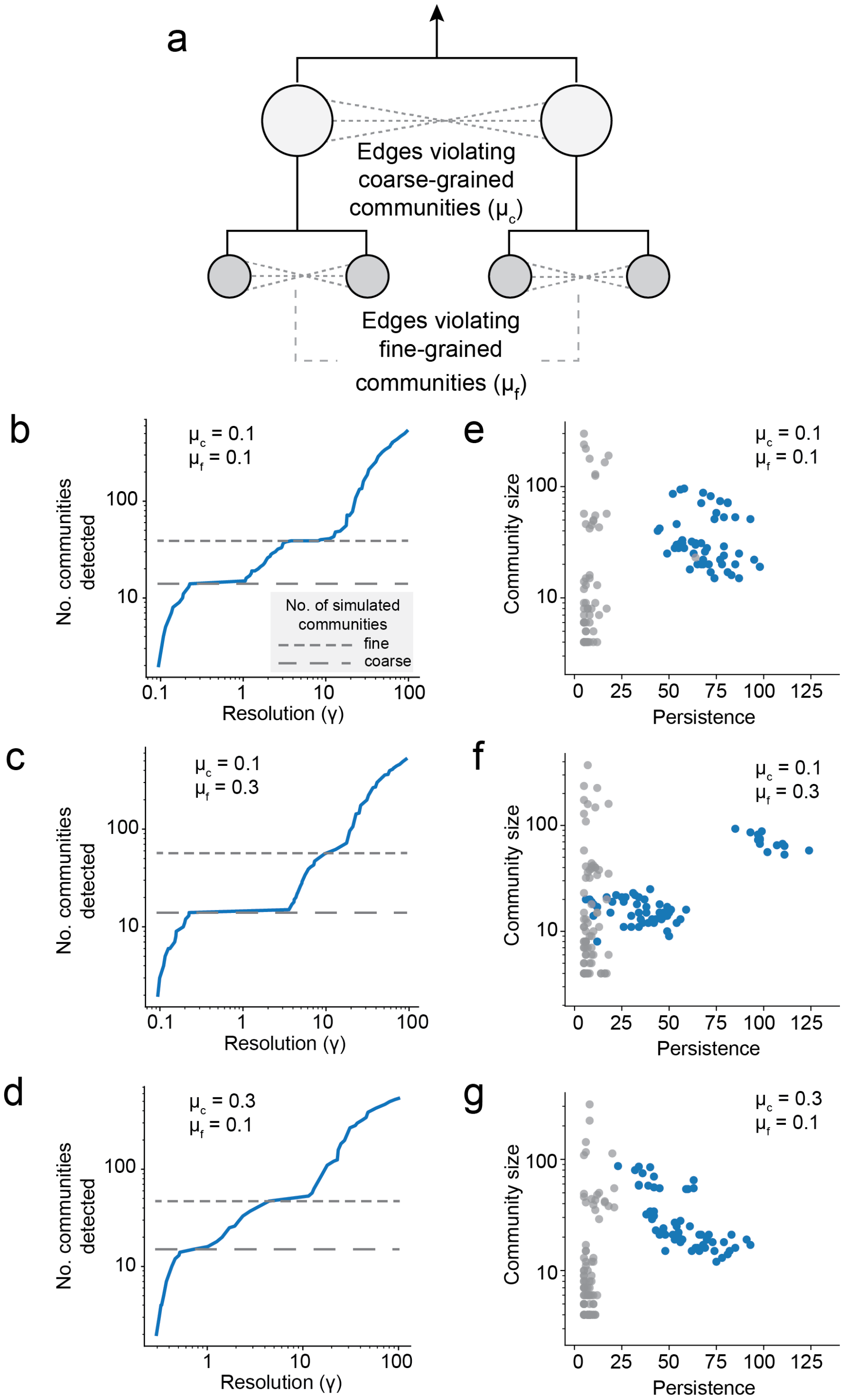

Fig. S1. Exploring simulated networks. a, The LFR generative model [1] was used to simulate networks with 1000 vertices and average degree 10 (**Methods**). The simulation included two layers of communities, "coarse" (10-20 communities, 50-100 vertices per community) and "fine" (25-200 communities, 5-40 vertices per community), with each fine community nested within a coarse community. Two "mixing parameters" μ_c and μ_f controlled the amount of noise, by setting the fraction of edges violating the coarse and fine community structures, respectively. **b-d,** HiDeF analysis of three simulated networks created with different mixing parameters: low balanced noise (b); increased noise in fine communities (c); and increased noise in coarse communities (d). Each plot shows the number of identified communities (y axis) as the resolution is progressively scanned (x axis). The number of communities increases with the resolution parameter, with plateaus matching the actual numbers of coarse and fine communities in the simulated network (dashed lines). Note that the sizes of the plateaus (i.e. the extent of community "persistence", see text) are affected by the mixing parameters. **e-g**, Companion plots to panels (b-d). Points represent identified communities, delineated by size (y axis) and persistence (x axis). Blue/gray point colors indicate a match/non-match to a true community in the simulated network (Jaccard similarity > 0.75). Note that when noise is low (e), the highest persistence communities correctly recover simulated communities with near-perfect accuracy, e.g. for persistence threshold >20.

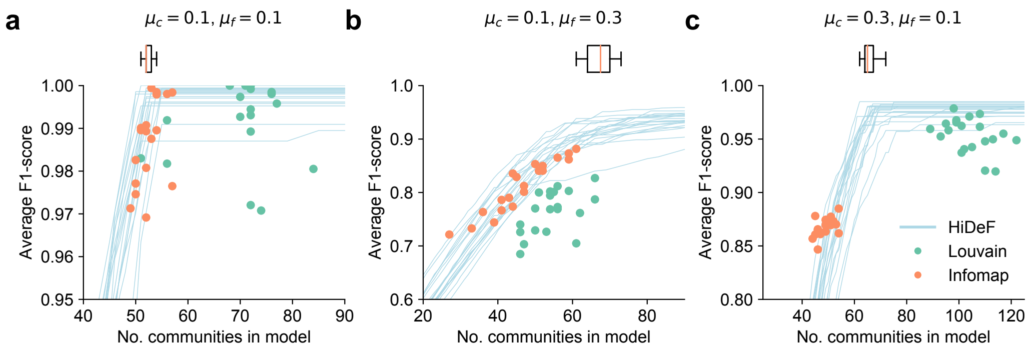

Fig. S2. Comparison of methods in recovery of simulated communities. HiDeF is compared with the Louvain and Infomap algorithms [2, 3], with Louvain and Infomap fixed at their default single resolutions (**Methods**). The three plots (**a-c**) compare the performance of the three algorithms in recovering simulated communities at different settings of the coarse/fine mixing parameters (see **Fig. S1**). The communities returned by HiDeF are ordered by persistence to evaluate the recovery of among the top *N* most persistent communities (by the average F1-score), whereas Louvain and Infomap generate results with fixed number of communities (green and orange points, respectively). The box plots on the top indicate the numbers of simulated communities. Each plot represents the results for 20 simulations. Note that HiDeF reached the maximum recovery when considering communities at a threshold equal to the number of simulated communities. Louvain and Infomap usually did not generate correct number of communities, and/or generate communities with worse agreements to simulated communities.

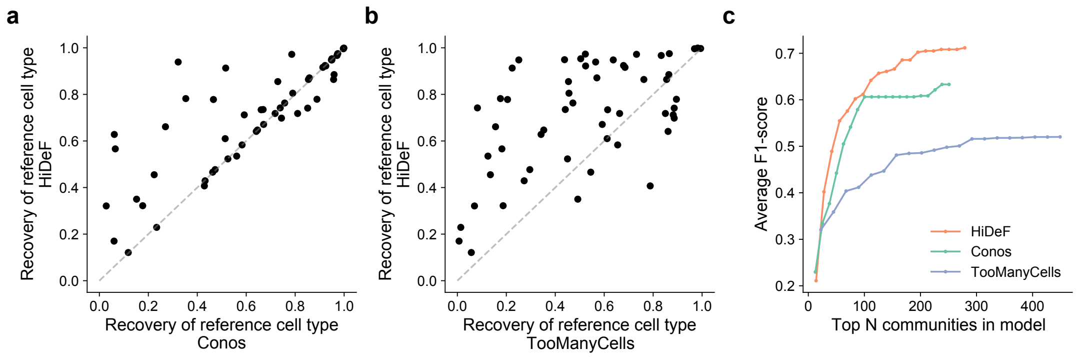

Fig. S3. Recovery of mammalian reference cell types from single-cell transcriptomes using the *Tabula Muris* droplet dataset [4]. Similar to **Fig. 2a-c**. **a-b,** Recovery of individual reference cell types by HiDeF (y axis) in comparison to Conos [5] or TooManyCells [6] (x axis of panels a or b, respectively). For each reference cell type (points), the extent of recovery is measured as the maximum F1-score of the set of reference cells with those of any detected community (see **Methods**). **c,** Reference cell types recovered (evaluated by the average F1-score) among the top *N* ranked cell communities. Communities are ranked in the descending order of score for each community detection tool (**Methods**).

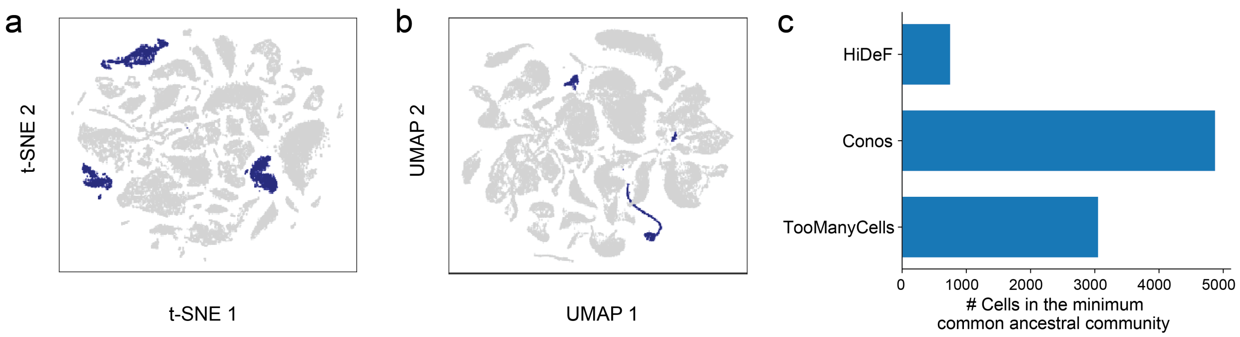

Fig. S4. Example cell types captured by HiDeF but not by other approaches. a, t-SNE projection of all cells, with the epidermal cell type highlighted (blue). **b,** UMAP projection of all cells, with the hepatocyte cell type highlighted (blue). **c**. Distances between astrocyte and neuron communities in the cell-type hierarchies generated by HiDeF, Conos, or TooManyCells. HiDeF identifies a specific super-community joining both cell types (<1000 cells), whereas such a specific community is not identified by Conos and TooManyCells.

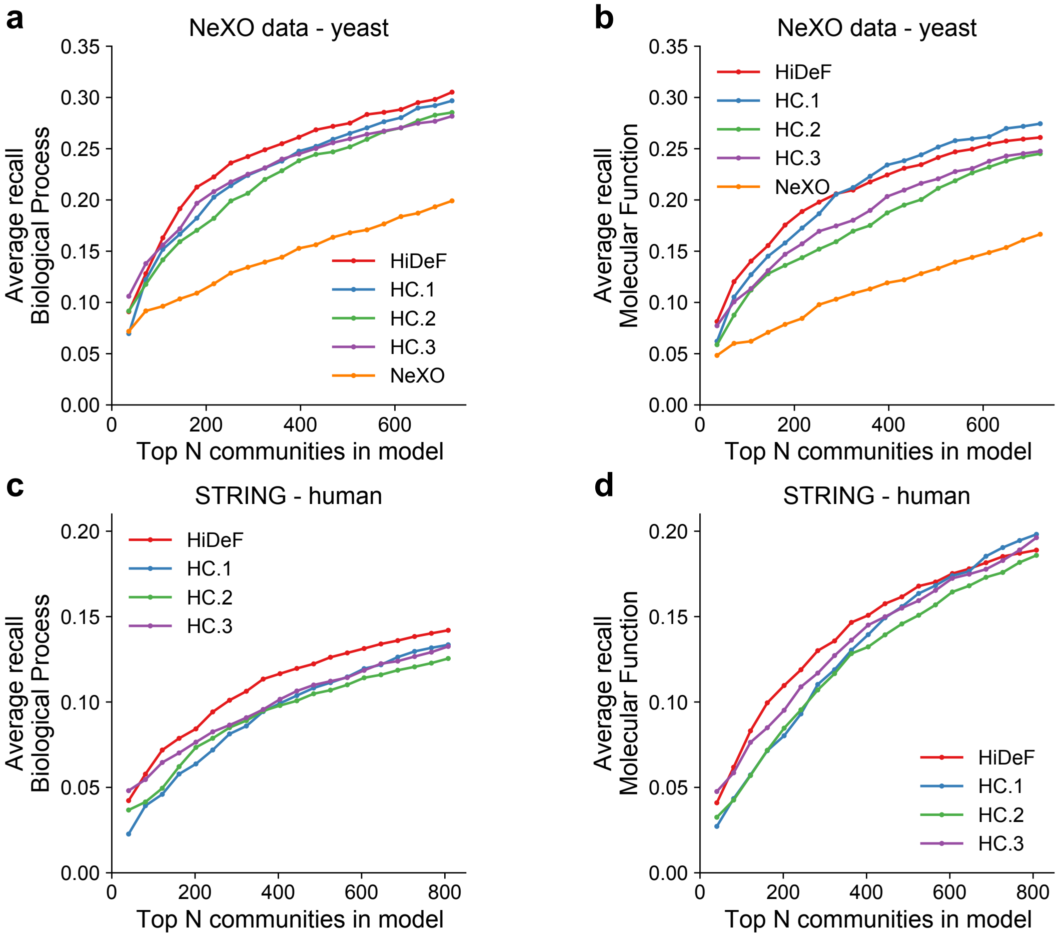

Fig. S5. Recovery of GO terms from community detection in protein networks. Similar to **Fig. 3a-b**. HiDeF and alternative methods were applied to build a hierarchy of protein communities from analysis of an integrated protein interaction network for budding yeast (Top: NeXO) or human (Bottom: STRING). The hierarchy of each method (colors) is scored by its recovery of GO terms (average F1 score; Left: Biological Process; Right: Molecular Function) as a function of the number of top-scoring protein communities examined. HC, Hierarchical Clustering following any of three protein pairwise distance functions (Mashup, DSD, and deepNF) [7-9].

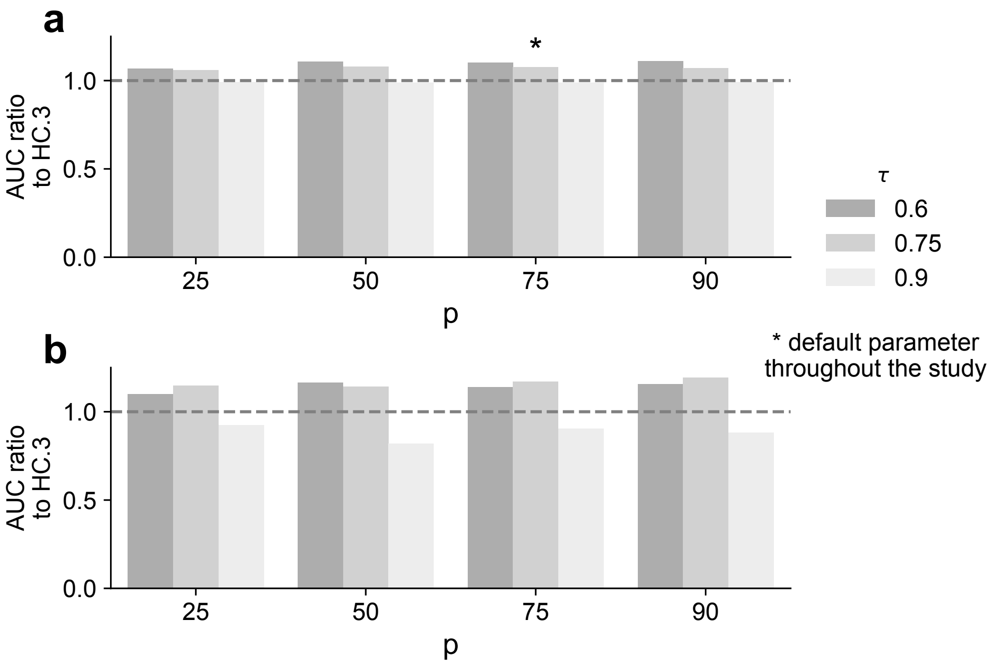

Fig. S6. Robustness of GO term recovery to the choice of parameters. **a**, Using the performance analysis depicted in **Fig. 3b**, the Area Under Curve (AUC) was computed for different sets of HiDeF parameters (p, τ). This AUC was compared to that of the best baseline tool, HC.3 (i.e. hierarchical clustering of pairwise distances generated by deepNF [9]) to generate an equal number of communities (**Methods**). Note the ratio HiDeF AUC / HC.3 AUC is usually higher than 1, indicating the favorable performance of HiDeF except for very high values of the τ parameter. As per **Fig. 3b**, the analysis was undertaken using the STRING network and the GO Cellular Component branch. **b**, Similar analysis with subsampling of network edges (in which a random 10% of network edges are removed prior to community detection at each resolution).

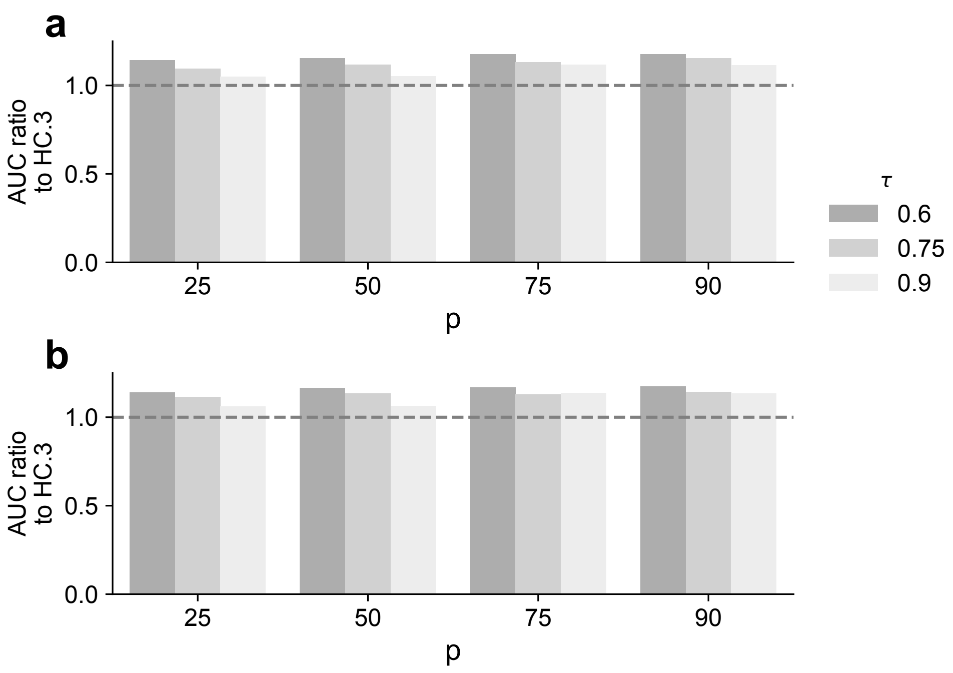

Fig. S7. Combining HiDeF and network embedding further improves recovery of GO terms. HiDeF was applied to an SNN graph based on the deepNF embedding (see **Methods**). Other settings of this analysis are identical to that in **Fig. S6**. Note that the performance of recovering GO terms in the Cellular Component branch is now better than HC.3 under all tested parameter settings of HiDeF.

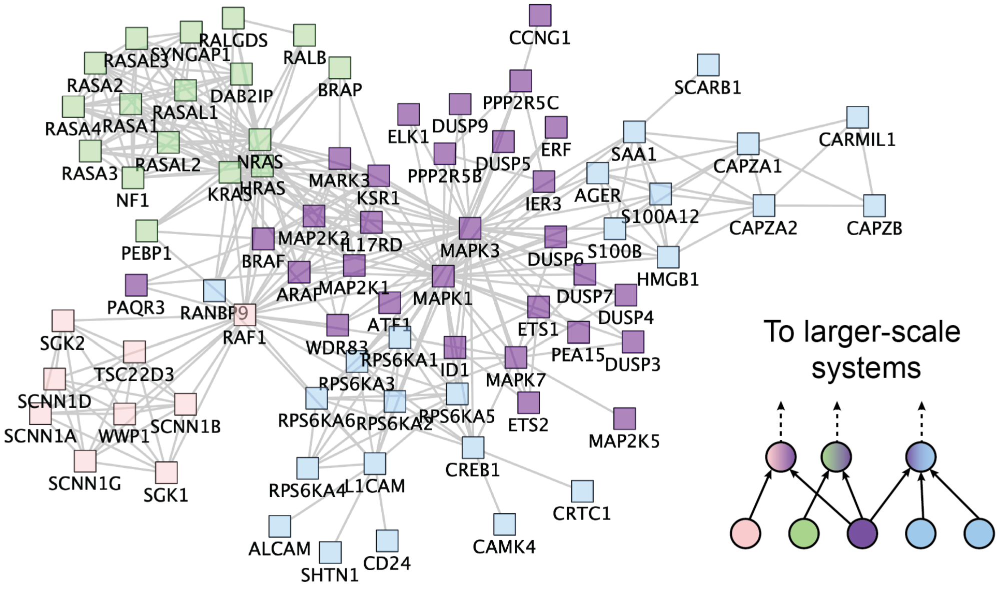

Fig. S8. Convergence of communities into multiple super-systems. A community of mitogen-activated protein kinases and dual-specificity phosphatases (purple, center) participates in three distinct larger communities involving separate functions related to RAS pathways (green), sodium channels (pink), and acting capping (blue). The corresponding hierarchical relationships of these communities are depicted at lower right. The source network is the Reactome [10].

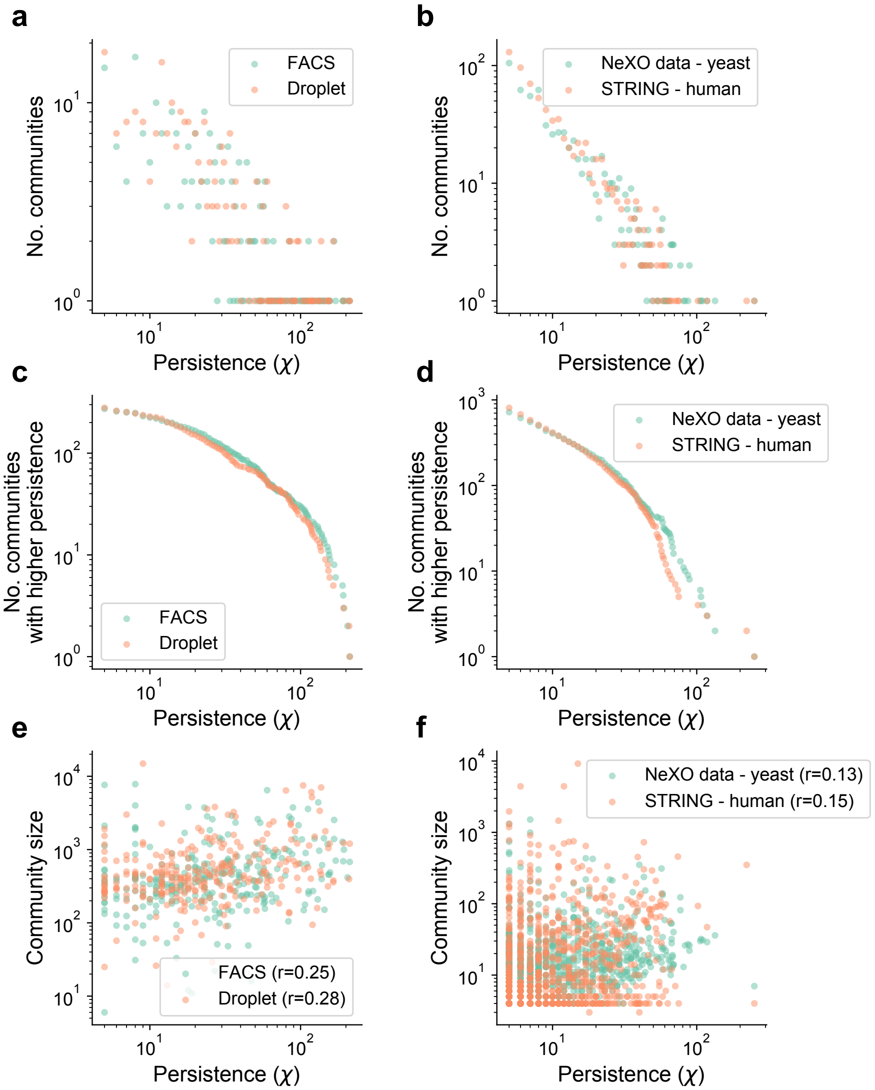

Fig. S9. Persistence of HiDeF communities. a-b, The number of communities (y axis) at each value of persistence (x axis). **c-d,** The number of communities with higher persistence (y axis) than a given threshold (x axis). **e-f,** Scatterplots of community size (y axis) versus persistence (x axis). The left column characterizes the single-cell transcriptomics data (**Fig. 2; Fig. S3**). The right column (panel b, d, f) characterizes the yeast and human protein-protein interaction datasets (**Fig. 3a, b**).

References

1. Lancichinetti A, Fortunato S, Radicchi F: **Benchmark graphs for testing community detection algorithms.** *Phys Rev E Stat Nonlin Soft Matter Phys* 2008, **78:**046110.

2. Blondel VD, Guillaume J-L, Lambiotte R, Lefebvre E: **Fast unfolding of communities in large networks.** *J Stat Mech* 2008, **2008:**P10008.

3. Rosvall M, Bergstrom CT: **Maps of random walks on complex networks reveal community structure.** *Proc Natl Acad Sci U S A* 2008, **105:**1118-1123.

4. Tabula Muris C, Overall c, Logistical c, Organ collection and p, Library preparation and s, Computational data a, Cell type a, Writing g, Supplemental text writing g, Principal i: **Single-cell transcriptomics of 20 mouse organs creates a Tabula Muris.** *Nature* 2018, **562:**367-372.

5. Barkas N, Petukhov V, Nikolaeva D, Lozinsky Y, Demharter S, Khodosevich K, Kharchenko PV: **Joint analysis of heterogeneous single-cell RNA-seq dataset collections.** *Nat Methods* 2019.

6. Schwartz GW, Zhou Y, Petrovic J, Fasolino M, Xu L, Shaffer SM, Pear WS, Vahedi G, Faryabi RB: **TooManyCells identifies and visualizes relationships of single-cell clades.** *Nat Methods* 2020.

7. Cho H, Berger B, Peng J: **Compact Integration of Multi-Network Topology for Functional Analysis of Genes.** *Cell Syst* 2016, **3:**540-548.e545.

8. Cao M, Zhang H, Park J, Daniels NM, Crovella ME, Cowen LJ, Hescott B: **Going the distance for protein function prediction: a new distance metric for protein interaction networks.** *PLoS One* 2013, **8:**e76339.

9. Gligorijevic V, Barot M, Bonneau R: **deepNF: deep network fusion for protein function prediction.** *Bioinformatics* 2018, **34:**3873-3881.

10. Fabregat A, Jupe S, Matthews L, Sidiropoulos K, Gillespie M, Garapati P, Haw R, Jassal B, Korninger F, May B, et al: **The Reactome Pathway Knowledgebase.** *Nucleic Acids Res* 2018, **46:**D649-D655.
