# Supplementary material for: HiDeF: identifying persistent structures in multiscale ‘omics data
Source: Genome Biol. 2021 Jan 7;22:21. doi: 10.1186/s13059-020-02228-4 (PMC7789082; doi:10.1186/s13059-020-02228-4)
Supplement: Supplementary file 2 — Additional file 2. Review history. [file 13059_2020_2228_MOESM2_ESM.docx]

Review History

**First round of review**

**Reviewer 1**

**Were you able to assess all statistics in the manuscript, including the appropriateness of statistical tests used?**

Yes, and I have assessed the statistics in my report.

**Comments to author:**

The manuscript presented by Zheng, Zhang and colleagues describes a new method, HiDeF, for detecting complex structures in biological data. The method applies existing algorithms for the network community detection and borrows the concept of "persistent homology" from the algebraic topology theory, in order to detect the intrisic structure of data that is conserved across different scales. Overall this study is well constructed and presented. A comparison with other methods is provided with real and simulated datasets in the field of single-cell transcriptomics (scRNA-seq) and protein-protein interactions.

Below my comments:

1. I believe the connection with the "persistent homology" theory is interesting and makes clearer the idea behind the method as shown in the full Tabula Muris data (Figure 2d). However, I think the application of the tool in a more specific structured data is relevant and would give more value to this tool. There are many available large-scale datasets in which hierarchical structures are known and already identified by different types of algorithms. For example, the application of the tool to a large PBMC dataset, in which different subtypes of CD4+ or CD8+ T cells (e.g. naive/memory or even more subtypes like T helper, T helper 17, T regulatory cells, etc..) are identified, would be ideal to benchmark the specifity of the tool in detecting subtle cell subtypes.

Something similar is shown in figure 2e, but the inclusion of cells from different tissues and the identification of new putative cell types at "depth 4" is not fully convincing and difficult to demonstrate.

2. Why the results from the Tabula Muris droplet datasets are not shown? Do authors think their tool works better with data generated by more sensitive scRNA-seq protocols? Authors should address this point by comparing the performance of their method in data generated with different protocols (both plate-based and droplet-based).

3. There are very few information about the data input. Are authors using all genes to make the graph? Are data normalized and highly variable genes detected before the application of the Louvain algorithm?

4. Conos was specifically designed for batch correction in case of data coming from different protocols or samples with different genetic backgrounds. How does HiDeF work under these challenging conditions?

5. Some of the most common tools in the single cell community (e.g. Seurat and Scanpy) use similar graph-based algorithms to detect cell subpopulations. Why these tools were not considered by the authors in the comparison against their tool?

===

Please consider the following questions:

- Are the methods appropriate to the aims of the study, are they well described, and are necessary controls included? If not, please specify what is required.

- Are the conclusions adequately supported by the data shown? If not, please explain

- Are sufficient details provided to allow replication and comparison with related analyses that may have been performed? If not, please specify what is required.

- Does the work represent a significant advance over previously published studies?

- Is the paper of broad interest to others in the field, or of outstanding interest to a broad audience of biologists?

**Reviewer 2**

**Were you able to assess all statistics in the manuscript, including the appropriateness of statistical tests used?**

Yes, and I have assessed the statistics in my report.

**Comments to author:**

In this manuscript, Zheng and colleagues present HiDeF to detect structure from omics data, HiDeF leverages the topology concept of "persistent homology" and is able to identify, from various biological data, structure robust to the scale or resolution of analysis.

Structure detection is valuable for the analysis and interpretation of many omics data we have collected nowadays. The Ideker lab has developed and been maintaining the widely-adopted network construction tool Cytoscape (PMID: 14597658, with >19,000 google scholar citations). Having HiDeF also available through the Cytoscape platform will definitely help dissemination of the method. Together with other of HiDeF's desirable properties (detailed below), I consider HiDeF to have high impact potential.

The manuscript is well-written with methods well justified and clearly presented. There are many merits of this work. I will just name two major ones: (1) the method used is novel in this context. The mathematical foundation behind HiDeF, the concept of persistent homology, is solid. The idea has been recently used for network inference in brain but HiDeF employs the concept for more general purposes here. (2) The authors carefully conducted both simulation studies as well as real data applications (notably to two rather distinct types of biological data) with results clearly explained. They authors show rather compelling evidences that HiDeF outperforms state-of-the-art methods (e.g., Conos and TooManyCells published this year or last year in Nature Methods) and generates biologically sensible results at a spectrum of scale/resolutions.

I have only the following minor comment/suggestion. The authors claim that HiDeF can detect robust structures in data at **all** scales simultaneously. This is almost too good to be true. My hunch is that the persistent threshold 𝜒 could influence the performance, potentially qualitatively. It would be desirable if the authors can further comment on that.

**Reviewer 3**

**Were you able to assess all statistics in the manuscript, including the appropriateness of statistical tests used?**

Yes, and I have assessed the statistics in my report.

**Comments to author:**

The authors proposed HiDeF which adopted persistent homology to identify robust structures in multi-scale data simultaneously.

The proposed method is straightforward and works well. Overall, the paper is well-written and the method is promising.

The work can be improved by addressing the following questions.

Major

1. For the evaluation of HiDeF, it seems that evaluation metrics used in the paper are problem-specific. Could you define/use some

standard metrics for evaluating the performance of multi-scale structure identification methods?

2. - p.6. "... HiDeF captured knowledge in GO more significantly than ... standard hierarchical clustering of pairwise protein distances calculated by two recent approaches ... "

- Mashup was published in 2016 and DSD in 2013. The authors should explain why these two methods are chosen since there are some new methods that can generate protein embeddings from networks.

For example, DeepNF.

Minor

1. In Supplementary Figure 4, you only provide results of Biological Process and Molecular Function. How about Cellular Component?

2. In "Protein-protein interaction networks", you use DDOT package to create GO reference for human proteins. Why not directly from GO annotations in the UniProt knowledgebase?

**Authors Response**

**Point-by-point responses to the reviewers’ comments:**

Reviewer #1:

The manuscript presented by Zheng, Zhang and colleagues describes a new method, HiDeF, for detecting complex structures in biological data. The method applies existing algorithms for the network community detection and borrows the concept of "persistent homology" from the algebraic topology theory, in order to detect the intrisic structure of data that is conserved across different scales. Overall this study is well constructed and presented. A comparison with other methods is provided with real and simulated datasets in the field of single-cell transcriptomics (scRNA-seq) and protein-protein interactions.

*We thank the reviewer for the positive comments.*

Below my comments:

I believe the connection with the "persistent homology" theory is interesting and makes clearer the idea behind the method as shown in the full Tabula Muris data (Figure 2d). However, I think the application of the tool in a more specific structured data is relevant and would give more value to this tool. There are many available large-scale datasets in which hierarchical structures are known and already identified by different types of algorithms. For example, the application of the tool to a large PBMC dataset, in which different subtypes of CD4+ or CD8+ T cells (e.g. naive/memory or even more subtypes like T helper, T helper 17, T regulatory cells, etc..) are identified, would be ideal to benchmark the specificity of the tool in detecting subtle cell subtypes.

Something similar is shown in figure 2e, but the inclusion of cells from different tissues and the identification of new putative cell types at "depth 4" is not fully convincing and difficult to demonstrate.

*We appreciate the reviewer’s positive comment on our analysis of the Tabula Muris data. We chose Tabula Muris since it is a sophisticated dataset with reference cell populations annotated on many different levels/scales, which in previous methods papers had created a challenging application for development of new scRNAseq clustering algorithms. We agree with the reviewer that demonstrating the application of our HiDeF approach to another structured dataset, e.g. PBMCs, would add value. Of the scRNAseq datasets relating to PBMCs, we found that PBMC-68k [1] includes the largest number of gold-standard reference cell populations. We note that even for this dataset there are only 11 reference cell types, and they are not organized hierarchically according to the reviewer’s expectation. In particular, the reference cell types are CD8+ Cytotoxic T, CD8+/CD45RA+ Naive Cytotoxic, CD56+ NK, CD4+/CD25 T Reg, CD19+ B, CD4+/CD45RO+ Memory, CD14+ Monocyte, Dendritic, CD4+/CD45RA+/CD25- Naive T, CD34+, and CD4+ T Helper. In contrast, the Tabula Muris dataset that we included in the original submission includes over 130 reference cell type annotations. We also surveyed more recent studies with PBMC data, for instance a dataset by Ding et al. [2] generated by six different single-cell sequencing platforms, but we found that reference cell type annotations were also sparse in those studies (i.e. Ding et al. defined only nine reference cell types).*

*Thus, our survey of the published data suggests that known blood cell type annotations are not as readily available as the reviewer had reasonably expected. We agree that construction of a deeper hierarchical reference map of blood cell types would be very useful, but this would require an extensive survey of known gene markers, expression analysis, and domain knowledge, which is clearly beyond the scope of this work. If indeed we have missed some key PBMC dataset in the literature with the deeper hierarchical annotation expected by the reviewer, we would be happy to analyze it. In the meantime, we feel the very large number of reference cell types in the Tabula Muris dataset, including PBMC cell types, makes it without question the correct reference we should be using as a benchmark.*

2. Why the results from the Tabula Muris droplet datasets are not shown? Do authors think their tool works better with data generated by more sensitive scRNA-seq protocols? Authors should address this point by comparing the performance of their method in data generated with different protocols (both plate-based and droplet-based).

*We had in fact analyzed the Tabula Muris droplet datasets, but the results did not make the submitted version due to space considerations. We have put back this analysis as Supplementary Figure 3. The results support the same conclusions we made with Tabula Muris FACS data.*

3. There are very few information about the data input. Are authors using all genes to make the graph? Are data normalized and highly variable genes detected before the application of the Louvain algorithm?

*For the Tabula Muris data, we applied HiDeF directly to the SNN graphs generated by the Tabula Muris consortium, which are publicly available. Data normalization and selection of highly variable genes are identical to what was described in the original Tabula Muris paper [3]. We have added these and other details to the Methods section to clarify matters.*

4. Conos was specifically designed for batch correction in case of data coming from different protocols or samples with different genetic backgrounds. How does HiDeF work under these challenging conditions?

*We appreciate this helpful comment. The reviewer is right that Conos was designed for batch correction and integrating different samples, which is not the focus of HiDeF. The batch correction functionality of Conos relies on generating a graph in which edges can link cells within or across samples. We do not present HiDeF as a tool to perform batch correction. However, we think that valuable insights about the structure of data can also be obtained if HiDeF is applied to a complex graph generated by batch correction tools like Conos. We will work on that aspect in a future development of HiDeF.*

5. Some of the most common tools in the single cell community (e.g. Seurat and Scanpy) use similar graph-based algorithms to detect cell subpopulations. Why these tools were not considered by the authors in the comparison against their tool?

*We thank the reviewer for this helpful comment. Unlike HiDeF, Seurat and Scanpy do not consider integration of results generated at different resolution parameters, and they do not have the concept of persistence for the identified communities. Therefore, we considered comparison to TooManyCells and Conos, as alternatives. The ability to identify multiscale communities was the main feature of TooManyCells. The main feature of Conos was batch correction and integration of different datasets, but the authors also showed results of multiscale community identification in their paper as one strength of Conos. We added the following text to the section “single-cell RNA-seq data” in Methods.*

*“We chose to compare with these methods because their ability to identify multiscale communities was either the main advertised feature or had been shown to be a major strength”.*

Reviewer #2:

In this manuscript, Zheng and colleagues present HiDeF to detect structure from omics data, HiDeF leverages the topology concept of "persistent homology" and is able to identify, from various biological data, structure robust to the scale or resolution of analysis.

Structure detection is valuable for the analysis and interpretation of many omics data we have collected nowadays. The Ideker lab has developed and been maintaining the widely-adopted network construction tool Cytoscape (PMID: 14597658, with >19,000 google scholar citations). Having HiDeF also available through the Cytoscape platform will definitely help dissemination of the method. Together with other of HiDeF's desirable properties (detailed below), I consider HiDeF to have high impact potential.

The manuscript is well-written with methods well justified and clearly presented. There are many merits of this work. I will just name two major ones: (1) the method used is novel in this context. The mathematical foundation behind HiDeF, the concept of persistent homology, is solid. The idea has been recently used for network inference in brain but HiDeF employs the concept for more general purposes here. (2) The authors carefully conducted both simulation studies as well as real data applications (notably to two rather distinct types of biological data) with results clearly explained. They authors show rather compelling evidences that HiDeF outperforms state-of-the-art methods (e.g., Conos and TooManyCells published this year or last year in Nature Methods) and generates biologically sensible results at a spectrum of scale/resolutions.

*We appreciate the reviewer’s many positive comments on the merits of our work.*

I have only the following minor comment/suggestion. The authors claim that HiDeF can detect robust structures in data at **all** scales simultaneously. This is almost too good to be true. My hunch is that the persistent threshold could influence the performance, potentially qualitatively. It would be desirable if the authors can further comment on that.

*We agree with the reviewer that the persistence threshold (chi) can influence the performance, and we have added further details and discussion of this relationship in the main text. We had in fact already included relevant experiments in the manuscript, which the new Methods text now better references and highlights. In particular, in Figs. 2c, 3a, 3b, and Supplementary Fig. 3, the x-axis reads "Top N communities in model”. For HiDeF, the means of selecting the top N communities was exactly to vary the persistence threshold (the chi parameter). Decreasing N leads to fewer communities in the model, accomplished by running the HiDeF program under higher persistence thresholds.*

*In the legend of Fig. 2c, we wrote "communities are ranked in descending order of score for each community detection tool (Methods)", and in Methods, we wrote "HiDeF communities were ranked by their persistence". We are sorry if the link between persistence threshold (the chi parameter) and the top N ranking procedure was not clear.*

*We have also added a supplementary figure (Supplementary Fig. 9) to show the distribution of community persistence, and the relationships between persistence and community sizes in the datasets used in our major analyses (Fig 2a-c, Fig. 3a-b). One can see that the community persistence only moderately correlates with community size, which means at more stringent thresholds of χ, the scales of the identified communities remain diverse.*

*To further clarify these matters, we have added the following sentences to the Methods section:*

*“Note that since χ is a threshold of minimum persistence, the results under a larger value of χ' can be produced by simply removing communities with persistence lower than χ' (Figs. 2c, 3a-b; Supplementary Figs 2, 3c, 5).*

*“The persistence of communities only moderately correlates with community sizes, with the consequence that different choices of persistent threshold χ do not strongly favor structures at particular scales (Supplementary Fig. 9)”*

Reviewer #3:

The authors proposed HiDeF which adopted persistent homology to identify robust structures in multi-scale data simultaneously.The proposed method is straightforward and works well. Overall, the paper is well-written and the method is promising.

*We thank the reviewer for the positive comments.*

The work can be improved by addressing the following questions.

Major

1. For the evaluation of HiDeF, it seems that evaluation metrics used in the paper are problem-specific. Could you define/use some standard metrics for evaluating the performance of multi-scale structure identification methods?

*For all challenge problems considered in the paper, we did in fact use the Jaccard similarity (between the inferred and reference hierarchy) as a common evaluation metric. It is possible however that the reviewer refers to the different thresholds on Jaccard similarity we used to distinguish “recovery” from “non-recovery” in the different problem domains. We agree that eliminating this parameter in evaluating the performance of multi-scale structure identification will make our paper stronger. Other than Jaccard similarity, most popular evaluation metrics, such as Rand Index and Normalized Mutual Information, are defined for non-overlapping clustering and are thus not appropriate for evaluating overlapping and multi-resolution clustering results. Instead, we have now implemented a version of the “Average F1-score” described in Yang and Leskovec, 2013 [4], which does not require a threshold parameter and does handle overlapping clusters. We have now replaced the major figures (Figs. 2c, 3a-b, Supp Figs. 2, 3c, 5, 6) of the paper with the results using this metric, all of which reach the same conclusions. The details of this metric are now explained in Supplementary Methods.*

2. - p.6. "... HiDeF captured knowledge in GO more significantly than ... standard hierarchical clustering of pairwise protein distances calculated by two recent approaches ... " - Mashup was published in 2016 and DSD in 2013. The authors should explain why these two methods are chosen since there are some new methods that can generate protein embeddings from networks. For example, DeepNF.

*To address the concern, we have now added a comparison to DeepNF [5], a method published in 2018 which creates node embeddings from network structures, followed by UPGMA hierarchical clustering. We have updated the results accordingly in Fig. 3 and Supplementary Figs. 5 and 6.*

*DeepNF had stronger performance than Mashup and DSD on the STRING cellular component task (Fig. 3b); We have therefore replaced Mashup (HC.1) with DeepNF (HC.3) in the comparison in Supplementary Fig. 6.*

*We observed some circumstances (e.g. tau = 0.9 with network perturbation) in which HiDeF performed worse than the combination of DeepNF and hierarchical clustering. We noted that in this comparison, HiDeF was directly applied to the original protein network (STRING) rather than the embedded representation. We found that applying HIDeF to a graph derived from the node embeddings generated by DeepNF further improved performance and the robustness to parameter choice. Thus, we have added a new figure (Supplementary Fig. 7) to show this new analysis of DeepNF + HiDeF.*

*These results show that HiDeF has high potential in identifying persistent structures in multiscale data when used in combination with network embedding techniques. On the other hand, network embedding methods tend to be computationally expensive, thus we believe that analyzing networks directly is an advantage of HiDeF and have elected to keep it as the default mode of the tool.*

Minor

1. In Supplementary Figure 4, you only provide results of Biological Process and Molecular Function. How about Cellular Component?

*The results of Cellular Component have been provided in Fig 3a-b.*

2. In "Protein-protein interaction networks", you use DDOT package to create GO reference for human proteins. Why not directly from GO annotations in the UniProt knowledgebase?

*We thank the reviewer for this comment. DDOT is a Python package and API we previously built for convenient access to the information in the Gene Ontology (GO) database, which does not affect the content of the GO data. We have clarified this rationale in the Methods:*

*“A reference GO for human proteins was downloaded from http://geneontology.org/ via an API provided by the DDOT package”.*

*References*

*1. Zheng GX, Terry JM, Belgrader P, Ryvkin P, Bent ZW, Wilson R, Ziraldo SB, Wheeler TD, McDermott GP, Zhu J, et al: Massively parallel digital transcriptional profiling of single cells. Nat Commun 2017, 8:14049.*

*2. Ding J, Adiconis X, Simmons SK, Kowalczyk MS, Hession CC, Marjanovic ND, Hughes TK, Wadsworth MH, Burks T, Nguyen LT: Systematic comparison of single-cell and single-nucleus RNA-sequencing methods. Nature biotechnology 2020:1-10.*

*3. Tabula Muris C, Overall c, Logistical c, Organ collection and p, Library preparation and s, Computational data a, Cell type a, Writing g, Supplemental text writing g, Principal i: Single-cell transcriptomics of 20 mouse organs creates a Tabula Muris. Nature 2018, 562:367-372.*

*4. Yang J, Leskovec J: Overlapping community detection at scale: a nonnegative matrix factorization approach. In Proceedings of the sixth ACM international conference on Web search and data mining. 2013: 587-596.*

*5. Gligorijevic V, Barot M, Bonneau R: deepNF: deep network fusion for protein function prediction. Bioinformatics 2018, 34:3873-3881.*

**Second round of review**

**Reviewer 1**

The authors have done significant work to improve the manuscript. They answered all questions and addressed problematic points I commented last time. The manuscript is now of a very high quality. Yet, a minor concern remain especially in the context of data input.

The authors claim that for the Tabula Muris data, the input was the shared nearest neighbor graph obtained from the Seurat analysis implemented in the original work. It is not clear if, in a similar context, users should always use such a graph to apply their tool or other input data can be also provided to HiDeF. Can authors clarify if the choice of the graph can impact on the HiDeF performance?

**Reviewer 3**

The authors have already addressed my questions in the revision. I recommend the paper to be accepted.
